# Effects of an Audiovisual Emotion Perception Training for Schizophrenia: A Preliminary Study

**DOI:** 10.3389/fpsyt.2021.522094

**Published:** 2021-05-05

**Authors:** Ji Woon Jeong, Hyun Taek Kim, Seung-Hwan Lee, Hyejeen Lee

**Affiliations:** ^1^Department of Psychology, Korea University, Seoul, South Korea; ^2^Department of Psychiatry, Ilsan-Paik Hospital, Inje University, Goyang, South Korea; ^3^Department of Psychology, Chonnam National University, Gwangju, South Korea

**Keywords:** schizophrenia, emotion perception, multimodal integration, audiovisual, training

## Abstract

Individuals with schizophrenia show a reduced ability to integrate facial and vocal information in emotion perception. Although emotion perception has been a target for treatment, no study has yet examined the effect of multimodal training on emotion perception in schizophrenia. In the present study, we developed an audiovisual emotion perception training and test in which a voice and a face were simultaneously presented, and subjects were asked to judge whether the emotions of the voice and the face matched. The voices were either angry or happy, and the faces were morphed on a continuum ranging from angry to happy. Sixteen patients with schizophrenia participated in six training sessions and three test sessions (i.e., pre-training, post-training, and generalization). Eighteen healthy controls participated only in pre-training test session. Prior to training, the patients with schizophrenia performed significantly worse than did the controls in the recognition of anger; however, following the training, the patients showed a significant improvement in recognizing anger, which was maintained and generalized to a new set of stimuli. The patients also improved the recognition of happiness following the training, but this effect was not maintained or generalized. These results provide preliminary evidence that a multimodal, audiovisual training may yield improvements in anger perception for patients with schizophrenia.

## Introduction

Impaired emotion perception in schizophrenia has been well-documented. Individuals with schizophrenia have deficits in identifying or discriminating emotions in others from facial expressions (1–3) and tone of the voice (4, 5). Furthermore, they have a reduced ability to integrate facial (visual) and vocal (auditory) emotional information. Specifically, patients with schizophrenia have been shown to not improve to the extent that healthy controls did in emotion recognition tasks when congruent audiovisual stimuli were presented (i.e., face and voice combined) compared to when audio or visual stimuli were presented alone (6, 7), and performed worse than healthy controls in the audiovisual condition [(8–10), cf. 11]. Deficits in the ability to perceive others' emotions are likely trait-like and serve as a risk marker for schizophrenia (12) and remain stable over the course of the illness (13). Moreover, these deficits have an impact on functional outcomes, such as social problem solving, social skills, and community functioning (14, 15). Therefore, addressing emotion perception in schizophrenia has been a target for treatment (16–18).

The most common intervention for emotion perception in schizophrenia is social-cognitive remediation based on Social Cognition and Interaction Training (19), which is a 24-session therapist-led group treatment targeting multiple domains of social cognition including emotion processing, social perception, theory of mind, and attribution bias. More focused intervention on emotion perception has also been developed and the most widely used program is Training of Affect Recognition (20). This program involves a 12-session manualized group training designed to improve facial emotion recognition by using strategies such as verbalization of typical features of emotional faces. More recently, emotion perception in a non-verbal, social context has been gauged by gesture behaviors in schizophrenia patients (21). Although these psychosocial interventions have yielded positive effects on emotion perception (22–24), no study has yet examined the effects of multimodal training on emotion perception in schizophrenia. Given that people with schizophrenia show diminished multimodal integration of facial and vocal emotional stimuli, and as emotion perception in natural circumstances typically requires simultaneous processing of both facial and vocal cues (13, 25, 26), it is important to develop and examine the effects of training that targets multimodal emotion perception abilities in schizophrenia.

In the present study, based on our prior work (27) we developed an audiovisual emotion perception training and test in which a voice and a face were simultaneously presented and trainees were asked to judge whether the emotion of the voice and the emotion of the face were the same or different. We selected angry and happy stimuli because they represent a bipolar dimension of valence (28), and angry and happy faces are the most distinctive with the least overlap in facial visual information processing (29). For the facial stimuli, we used morphed facial photos that varied on a continuum from angry to happy, which allowed us to create ambiguous expressions and to assess the level of difficulty of training. We aimed to report preliminary data regarding first training effects of this program by conducting a six-session intervention and one follow-up session in a group of patients with schizophrenia. We expected that although schizophrenia patients would perform worse than the healthy controls in accuracy of emotion recognition prior to the training, the patients would demonstrate significant improvement following the training. We also aimed to examine if the training has differential effects on the perceptions of angry and happy emotions based on previous findings that schizophrenia patients show more deficits in recognition of negative emotions than positive emotions [e.g., (30)] and expected that the training effects would be more pronounced for anger compared to happiness.

## Methods

### Subjects

Eighteen schizophrenia patients (SP) were recruited from a long-term care mental institution in South Korea. At the time of enrollment, all patients met the criteria for schizophrenia based on the Structured Clinical Interview for DSM-IV (SCID-IV) (31) and were on stable antipsychotic medication (risperidone or olanzapine). The psychotic symptoms were evaluated using the Positive and Negative Syndrome Scale (PANSS) (32). None of the patients had a history of central nervous system diseases (e.g., epilepsy or cerebrovascular accident), substance abuse, electroconvulsive therapy, mental retardation, head injury, or hearing impairment.

Twenty healthy controls (HC) were recruited via advertisements in local newspapers and flyers. Subjects were excluded if they had any neurological disorder, head injury, or personal or family history of psychiatric diseases. After the initial screening, potential HCs were interviewed using the SCID for Axis II Psychiatric Disorders and were excluded if they had any of these disorders (33). SP and HC groups were matched for age, sex, and education. Finally, 16 SP and 18 HC subjects participated to the end of the study. All subjects gave written informed consent before the experimental procedures commenced. The protocol was approved by the Institutional Review Board of Inje University Ilsan Paik Hospital.

### Audiovisual Emotion Perception Training and Tests

**Figure 1:**
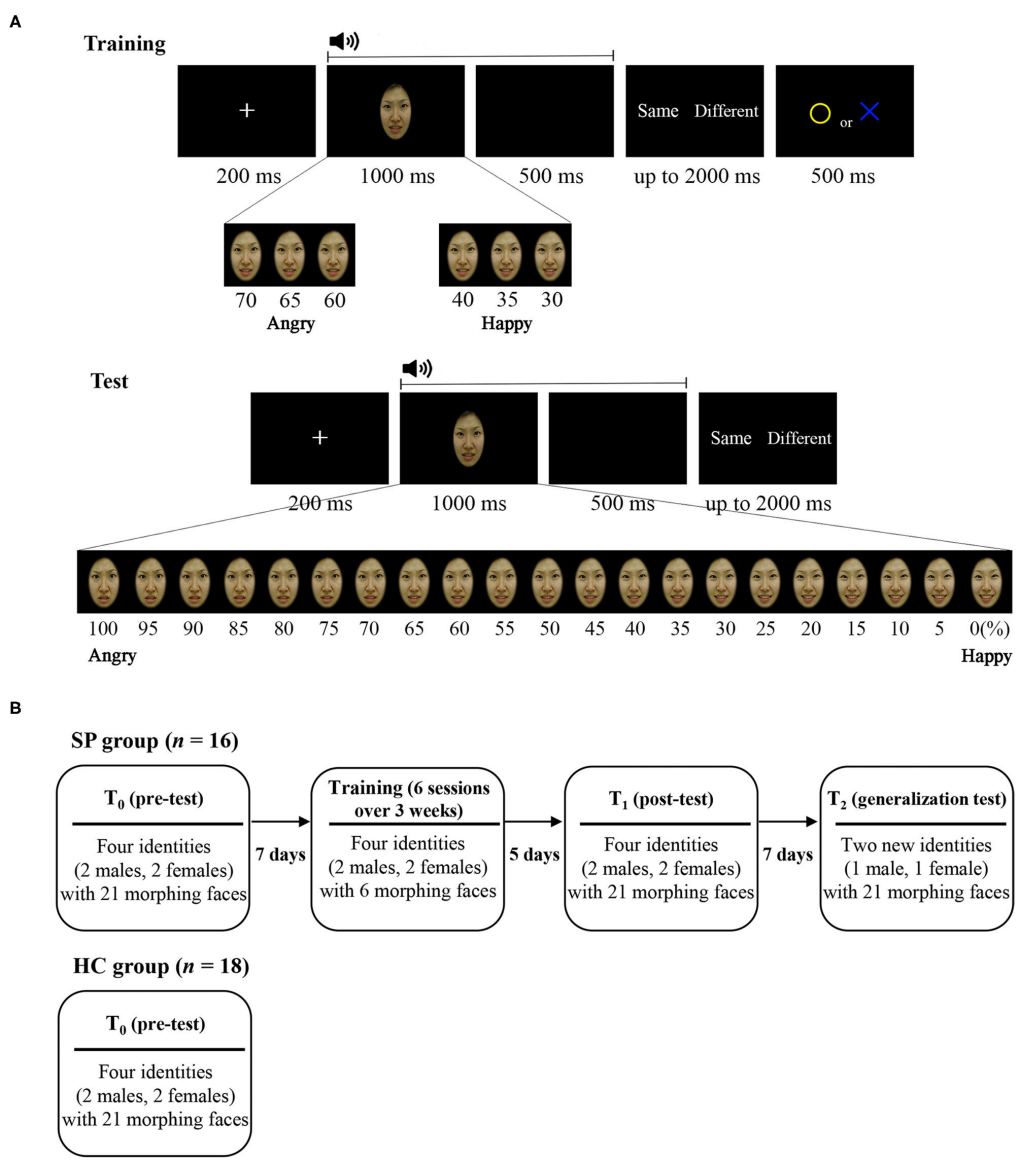
**(A)** Trial sequences and morphed images used at the training and test and **(B)** schematic illustration of the procedure. SP, schizophrenia patient; HC, healthy control.

As illustrated in Figure 1A, we developed the audiovisual emotion perception training and test. In this paradigm, each trial began with a fixation cross (200 ms) that was followed by the presentation of a morphed face (1,000 ms) and then a mask (black screen; 500 ms) while a voice was simultaneously delivered via earphones. Subjects were asked to judge whether the emotion of the face and the emotion of the voice were the same or different by pressing buttons that were counterbalanced across subjects. If subjects had correct responses, a yellow circle appeared, and if subjects had incorrect responses, a blue X appeared in the center of the screen for 500 ms. Trials of the training and the tests were the same except that feedback was given for subjects' responses only in the training.

The test, which was given prior to the training and after the training, consisted of 840 trials [21 morphed faces × 2 voice emotions × 4 identities (two males, two females) × 5 repetitions], of which 400 trials were congruent audiovisual stimulus pairs (i.e., angry face–angry voice, or happy face–happy voice), 400 trials were incongruent pairs (i.e., angry face–happy voice, or happy face–angry voice), and 40 trials had no correct answer (i.e., 50% morphed face–happy voice, or 50% morphed face–angry voice). The test that was given for the generalization test after the training consisted of 420 trials [21 morphed faces × 2 voice emotions × 2 identities (1 male, 1 female) × 5 repetitions], of which 200 trials were congruent, 200 trials were incongruent, and 20 trials had no correct answer.

The training consisted of 480 trials [6 morphed faces × 2 voice emotions × 4 identities (2 males, 2 females) × 10 repetitions], of which 240 trials were congruent and 240 trials were incongruent. For training purposes, only six images that were emotionally ambiguous from each continuum were used (i.e., 70, 65, 60, 40, 35, and 30% angry face).

For the facial stimuli, we created emotional morphed facial images using a computerized morphing program (Abrosoft FantaMorph version 5.4.1; Abrosoft, USA). We selected two prototypical photographs (i.e., angry and a happy face) of six identities (three males, three females) from a standardized facial stimulus set (Korea University Facial Expression Collection) (34) and created emotional morphed faces by merging the prototypical photographs of each identity in 5% steps, resulting in a morphing continuum in which a total of 21 faces with graded blending of the emotional facial features of the two faces. Finally, six angry–happy morphing continua for each six identities (three males, three females) were created. Among them, four morphing continua were used in the pre-training test session (T_0_), training session, and post-training test session (T_1_), and another two morphing continua (one male, one female) were used in the generalization test session (T_2_).

For the voice stimuli, we recorded valenced voices from amateur actors in a noise-free room. The actors were instructed to pronounce semantically neutral sentences (e.g., “stayed in the house”) either in an angry or happy voice. Sixteen voice stimuli (2 emotions × 2 sex × 4 sentences) were used in the training and tests. The mean length of the 16 voice stimuli was 1.06 s (*SD* = 59 ms).

### Procedure

Three days prior to the study, all subjects were tested on their ability to identify the valence of the auditory stimuli, which was necessary to perform the task. Forty auditory stimuli (2 emotions × 2 sex × 10 sentences) were presented, including the stimuli that were used in the study. Subjects were asked to determine whether the presented auditory stimulus represented anger or happiness. There was no difference in the accuracy between SP group (*M* = 38.688, *SD* = 1.537) and HC group (*M* = 39.105, *SD* = 1.729), *F*_(1, 33)_ =.561, *p* = 0.459.

The procedure of the training and tests is shown in Figure 1B. The SP group participated in a total of nine sessions: T_0_, six training sessions, T_1_, and T_2_. The T_0_ was to measure baseline that was conducted 7 days prior to the first training session. Training consisted of six sessions lasting over a period of 3 weeks. Each training session was divided into two blocks to reduce burden on subjects. Upon completing each block, subjects received tokens if they had scored 110% (for training sessions 1–3) or 120% (for training sessions 4–6) of the correct responses relative to T_0_. The number of tokens ranged from 1 to 12, and one token was ~1 US dollar. Five days after the final training session, the T_1_ was conducted to measure the effects of the training. To ascertain that the training effects would be generalizable, T_2_ was conducted using two faces that had not been previously used 7 days after T_1_. The HC group participated only in the T_0_.

### Statistical Analysis

Sensitivity for emotion perception was calculated as *d'* according to signal detection theory. The calculation of *d'* (sensitivity index) was based on the formula reported in the paper by Macmillan and Creelman (35). The sensitivity index provides the separation between the means of the signal and the noise distribution, in units of the standard deviation of the noise distribution. An estimate of *d'* can be found from measurements of the hit rate and false alarm rate. This is calculated as: *d'* = *Z*(hit rate) – *Z*(false alarm rate), where function *Z*(*p*), *p* ∈ [0,1], is the inverse of the cumulative Gaussian distribution. A correction of extreme proportions of hit and false-alarm rates was applied as proposed by Macmillan and Kaplan (36). A higher *d'* indicates that the signal is more readily detected.

In order to rule out any possible covariates, correlations between emotion perception (*d'*) and demographic/clinical characteristics of the patients, including age, sex, education, age of onset, two WAIS subtest scores, and PANSS scores (total score and five subscale scores), were analyzed using nonparametric correlational analyses (Spearman's rho).

Departure from normal distribution assumption was tested by the Shapiro–Wilk's test. Due to normality of data, descriptive statistics show means and standard deviations. To examine group differences in demographic and clinical information, paired-sample *t*-tests and chi-square test were conducted. To investigate pre-existing group difference, a mixed ANCOVA on *d'* was performed with Group (SP, HC) as a between-subjects factor, Emotion (anger, happiness) and Morphing (three levels; lv1: 100, 95, 90 and 10, 5, 0% of angry faces; lv2: 85, 80, 75 and 25, 20, 15% of angry faces; lv3: 70, 65, 60, 55, and 45, 40, 35, 30% of angry faces) as within-subjects factors, and premorbid IQ (WAIS Information, Vocabulary) as covariates. To evaluate the training and generalization effects, a repeated measures ANOVA on *d'* was performed with Emotion, Morphing, and Time (T_0_, T_1_, T_2_). Paired *t* tests were conducted if there were any significant results in the repeated measures ANOVA. The Bonferroni correction was used for pairwise comparisons and the Greenhouse–Geisser adjustment was used to correct for violations of sphericity.

## Results

### Characteristics of Subjects

Demographic and clinical data for the SP and HC groups are summarized in Table 1. There were no group differences in age, *t*_(32)_ = 0.112, *p* = 0.912, education, *t*_(32)_ = 0.489, *p* = 0.628, or sex ratio, χ^2^ = 0.133, *p* =0.744. However, there was a group difference in the WAIS Information subtest, *t*_(32)_ = 2.267, *p* = 0.030 and Vocabulary subtest, *t*_(32)_ = 2.957, *p* = 0.006. Therefore, analyses of group comparison used the WAIS Information and Vocabulary scores as covariates.

**Table 1:** Characteristics of the subjects.

**Variable**	**SP group (*n* = 16)**	**HC group (*n* = 18)**	**Group differences**
Age (years)	47.063 ± 7.104	47.333 ± 7.021	*t* = 0.112
Sex			χ^2^ = 0.133
Male	9 (56.25)	9 (50.00)	
Female	7 (43.75)	9 (50.00)	
Education (years)	11.938 ± 2.265	12.222 ± 0.943	*t* =.489
Premorbid IQ (WAIS)			
Information	12.438 ± 2.220	14.111 ± 2.083	*t* = 2.267*
Vocabulary	11.688 ± 2.056	13.667 ± 1.847	*t* = 2.957**
Duration of illness (years)	19 (7–34)	–	–
Onset age (years)	26.125 ± 6.355	–	–
Dosage of medication (CPZ equivalent, mg)	325.050 ± 425.500	–	–
Global symptom score (PANSS)			
Positive	21.500 ± 7.005	–	–
Negative	11.625 ± 4.425	–	–
Disorganized	17.500 ± 3.425	–	–
Excited	12.813 ± 3.103	–	–
Anxiety/depression	17.500 ± 4.066		–
Total	80.938 ± 5.133	–	–

*p < 0.05, **p < 0.01; WAIS, Wechsler Adult Intelligence Scale; CPZ, chlorpromazine; PANSS, Positive and Negative Syndrome Scale.

### Correlations Between Emotion Perception (*d'*) and Demographic and Clinical Characteristics

Correlations between emotion perception (*d'*) and all demographic and clinical characteristics of the SP group were not significant. These results indicate that demographic and clinical characteristics were not associated with emotion perception abilities in the patients.

### Pre-existing Group Differences

**Figure 2:**
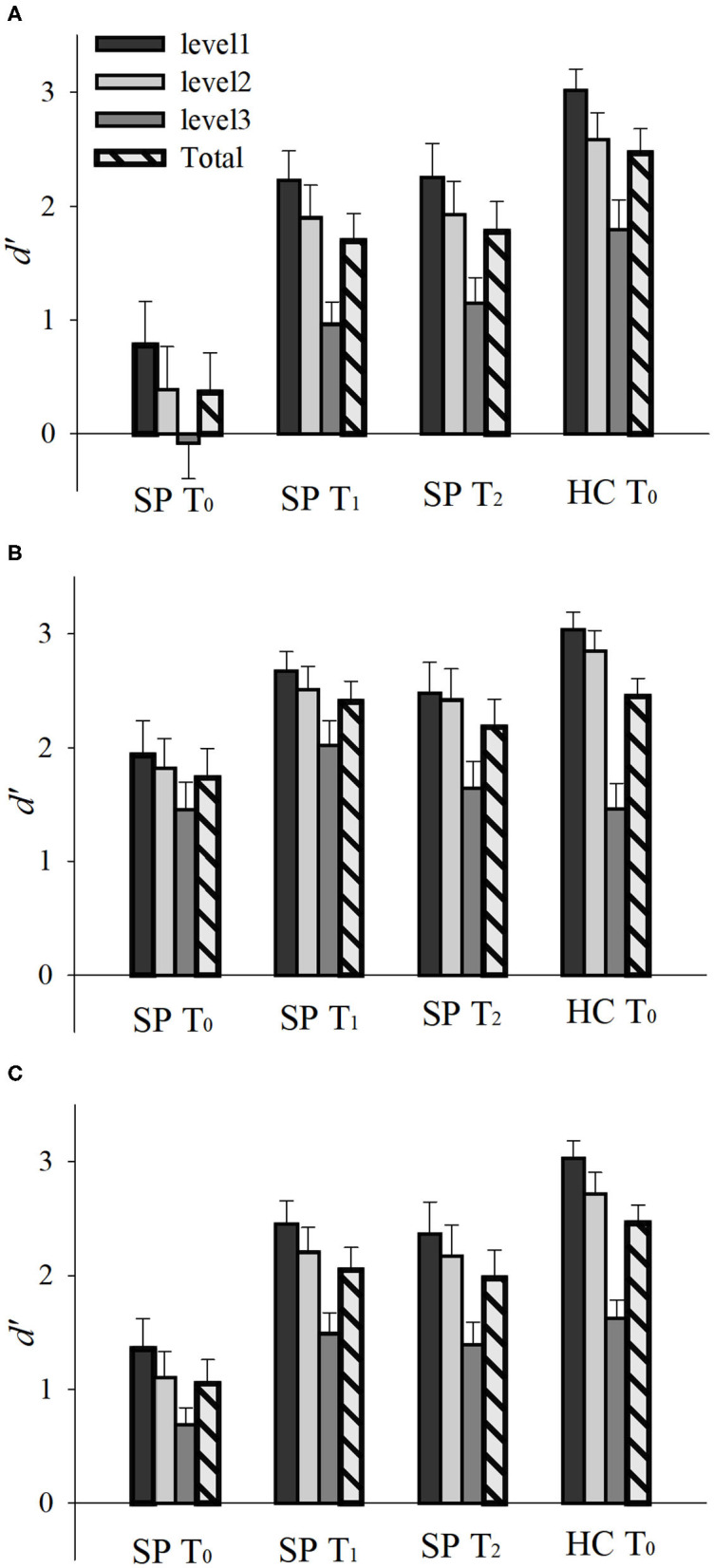
Emotion perception performances (*d'*) of the SP and HC according to time (T_0_, T_1_, and T_2_). Emotion perception performances for **(A)** anger, **(B)** happiness, and **(C)** total. *d'* = *Z*(hit rate) – *Z*(false alarm rate), where function *Z*(*p*), *p* ∈ [0,1]. Error bar indicates standard error. SP, schizophrenia patient; HC, healthy control. T_0_, pre-training session; T_1_, post-training session; T_2_, generalization session.

**Figure 3:**
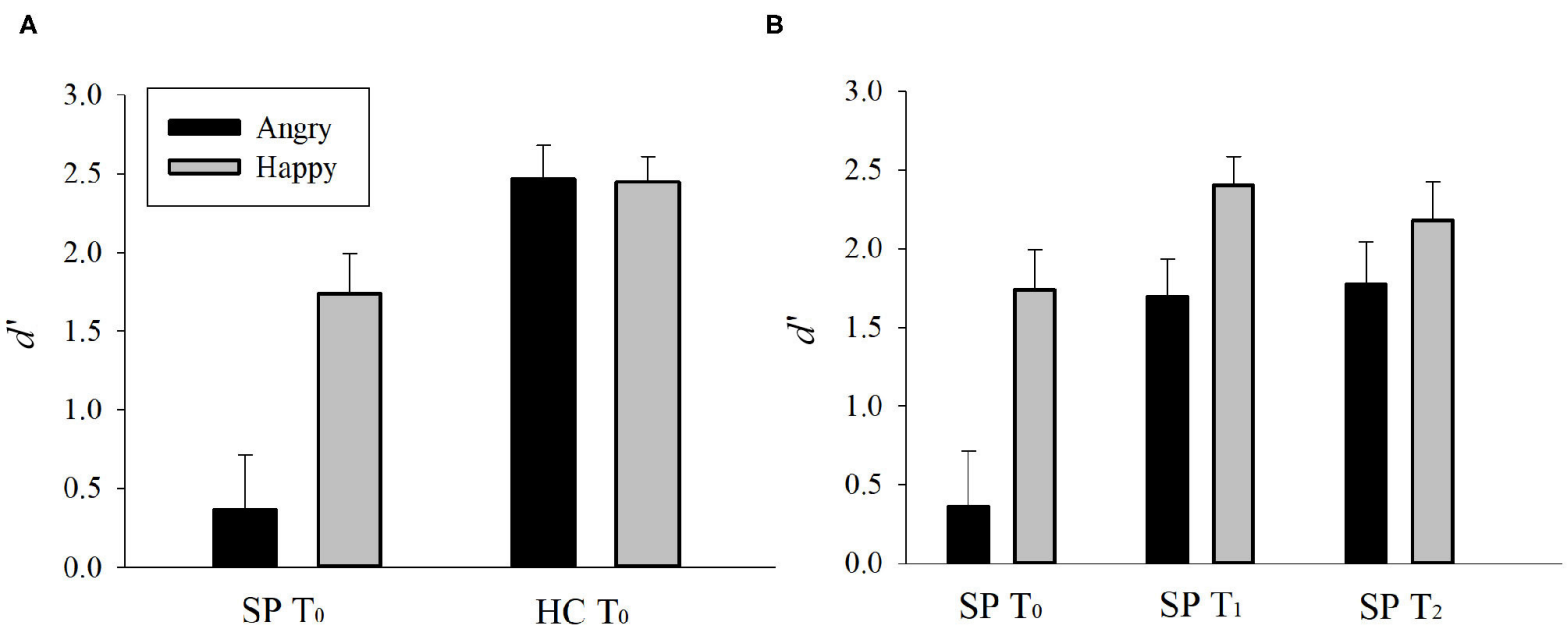
**(A)** Pre-existing group differences and **(B)** training and generalization effects in SP group. *d'* = *Z*(hit rate) – *Z*(false alarm rate), where function *Z*(*p*), *p* ∈ [0,1]. Error bar indicates standard error. SP, schizophrenia patient; HC, healthy control. T_0_, pre-training session; T_1_, post-training session; T_2_, generalization session.

Emotion perception performances (*d'*) of the SP and HC groups on T_0_ are presented in Figures 2, 3A. There was a main effect of Group, *F*_(1, 30)_ = 27.748, *p* < 0.001, $\eta_{p}^{2}$ = 0.480, such that the SP group performed worse than did the HC group. There was also an interaction effect of Group and Emotion, *F*_(1, 30)_ = 7.262, *p* = 0.011, $\eta_{p}^{2}$ = 0.195. The SP group performed worse than did the HC group both for anger, *t* = 5.232, *p* < 0.001 and happiness, *t* = 2.404, *p* = 0.022. These results indicate that prior to the training, the SP group performed worse than did the HC group in audiovisual perception of anger as well as happiness.

### Training and Generalization Effects in SP Group

Emotion perception performances (*d'*) of the SP group on T_0_, T_1_, and T_2_ are presented in Figures 2, 3B. There was a main effect of Time, *F*_(2, 30)_ = 15.336, *p* = 0.001, $\eta_{p}^{2}$ = 0.506, such that the performances of T_1_ and T_2_ were better than that of T_0_, *t*s > 3.796, *p*s < 0.002, and there was no difference between T_1_ and T_2_, *t* = 0.478, *p* = 0.640. There was also a main effect of Emotion, *F*_(1, 15)_ = 19.617, *p* < 0.001, $\eta_{p}^{2}$ = 0.567, such that the perception of happiness was better than that of anger. Furthermore, there was an interaction effect of Time and Emotion, *F*_(2, 30)_ = 3.406, *p* = 0.046, $\eta_{p}^{2}$ = 0.185. For anger, the performances of T_1_ and T_2_ were better than T_0_, *t*s > 4.335, *p*s < 0.001, and there was no difference between T_1_ and T_2_, *t* = 0.392, *p* = 0.701. For happiness, the performance of T_1_ was better than T_0_, *t* = 2.234, *p* = 0.041, but T_2_ was not different from T_0_, *t* = 1.243, *p* = 0.233. There was no difference between T_1_ and T_2_, *t* = 1.473, *p* = 0.161. These results indicate that the training improved and maintained the audiovisual perception for anger. However, the training improved but not maintained the audiovisual perception for happiness in the SP group.

## Discussion

This study provides preliminary evidence that the audiovisual emotion perception training we developed may be a promising method for improving emotion perception for people with schizophrenia. The results showed that prior to the training, the schizophrenia group performed significantly worse than did the control group in the recognition of both anger and happiness, and the recognition of anger was much worse than that of happiness. These findings are consistent with previous findings that show that, compared to healthy controls, schizophrenia patients were significantly worse at recognizing angry faces as well as happy faces (37–42). It is also in line with the extant literature suggesting that schizophrenia patients perform more poorly with negative emotions such as anger, fear, or sadness, compared to positive emotions such as happiness or joy [e.g., (30, 43–45)].

Following the training, the schizophrenia group showed significant improvement in recognizing anger. Specifically, compared to their performance during the pre-training, the patients performed better across all levels of morphing after the six sessions of training as well as at the follow-up session with a new set of stimuli. With respect to happiness, although the training increased the patients' accuracy, this effect was not maintained or generalized to a new set of stimuli. These results provide preliminary evidence that the training program may yield improvements in the multimodal recognition of anger and happiness and that the training effect may be more reliable and pronounced for anger perception.

The training effects that were observed might be attributable to cross-modal effects in which information in one modality influences the emotion perception in another modality (46–48). In particular, studies on cross-modal effects found that the less ambiguous modality has a greater impact; for example, the effect of voice became greater as the faces were more morphed (49) or ambiguous (25). Our paradigm had morphed faces on the angry–happy continuum whereas voices were distinctively angry or happy. Thus, it is possible that the auditory cues influenced and guided the visual cues in integrating multimodal cues in our training. Indeed, auditory information has been known to guide eye movements in audiovisual emotional processing, such that an emotional voice yielded longer and more frequent fixations on emotionally congruent faces (50–52) and drew more attention to salient facial features, which could help to improve emotion recognition in schizophrenia patients (11).

To our knowledge, this is the first study of a multimodal training program targeting the integration of vocal and facial emotional information. This program has several strengths. First, the training is relatively brief, self- and computer-administered, and free from confounding effects of group interactions as in other common treatment allowing a more targeted intervention. Second, it uses morphed, ambiguous faces, which have greater ecological validity than the prototypical faces that were used in previous studies, and enabled the training to have increasing levels of task difficulty. Third, it has a follow-up session with a new set of stimuli based on which the generalizability of the findings can be examined.

There are several limitations of the current study that warrant more control conditions to draw a conclusion. First, we did not have post-training data of the healthy control group, which limited the control for the effect of repetition and unspecific components of the training. A randomized controlled trial should be followed to confirm the efficacy of the program. Second, because the tests we used were modified from the training program, “training to the test” effect might have occurred. An independent test on multimodal emotion perception shall be needed to measure the training effects (53). Third, more categories of emotion are required to rule out the possibility that the patients learned to differentiate between the two emotions in the current study. Lastly, it will be important for future studies to investigate the extent to which the changes brought out by this training impact people's everyday life, particularly social functioning.

In conclusion, this study provides an initial evaluation of the effects of the audiovisual emotion perception program. Although more controlled research is needed, the current results provide preliminary evidence that the multimodal training of the audiovisual using faces and vocal information might improve the perception of anger in individuals with chronic schizophrenia. Future research with more rigorous designs and longer-term follow-up with functioning outcomes is needed to confirm the efficacy of the program.

## Data Availability Statement

The datasets generated for this study will not be made publicly available. We did not get the IRB approval on this.

## Ethics Statement

The studies involving human participants were reviewed and approved by Institutional Review Board of Inje University Ilsan Paik Hospital. The patients/participants provided their written informed consent to participate in this study.

## Author Contributions

HK and JJ conceived and designed the study. S-HL provided access to patients and JJ collected data. HL and JJ analyzed and interpreted the data, and wrote the manuscript. All authors read and approved the final manuscript.

## Conflict of Interest

The authors declare that the research was conducted in the absence of any commercial or financial relationships that could be construed as a potential conflict of interest.
